# Pollen analysis of Australian honey

**DOI:** 10.1371/journal.pone.0197545

**Published:** 2018-05-16

**Authors:** J. M. Kale Sniderman, Kia A. Matley, Simon G. Haberle, David J. Cantrill

**Affiliations:** 1 School of Earth Sciences, University of Melbourne, Parkville, Victoria, Australia; 2 Department of Archaeology and Natural History, College of Asia and the Pacific, Australian National University, Canberra, Australian Capital Territory, Australia; 3 Royal Botanic Gardens Victoria, South Yarra, Victoria, Australia; Universiti Sains Malaysia, MALAYSIA

## Abstract

Pollen analysis is widely used to verify the geographic origin of honeys, but has never been employed in Australia. In this study, we analysed the pollen content of 173 unblended honey samples sourced from most of the commercial honey producing regions in southern Australia. Southern Australian vegetation is dominated by *Eucalyptus* (Myrtaceae) forests and, as expected, most Australian honeys are palynologically dominated by *Eucalyptus*, while other important components include Myrtaceae taxa such as *Corymbia*/*Angophora* and the tribe Leptospermeae; plus Brassicaceae, *Echium*, *Macadamia*, and *Acacia*. An important feature of the honeys is the number of Myrtaceae pollen morphotypes per sample, which is generally high (mean = 4.6) compared to honeys produced outside of Australia, including *Eucalyptus* honeys produced in the Mediterranean region, and honeys produced in South America, which has its own rich indigenous Myrtaceae flora. In the latter regions, the number of Myrtaceae morphotypes is apparently generally ≤2. A high number of Myrtaceae morphotypes may be a feasible criterion for authenticating the origin of Australian honeys, since most Australian honey is produced by honey bees mainly working indigenous floral resources. Myrtaceae morphotype diversity is a convenient melissopalynological measure that could be applied even where detailed knowledge of the pollen morphology of the many component genera and species is absent. Palynological criteria developed in Europe for authenticating *Eucalyptus* honeys should not be relied upon for Australian honeys, since those criteria are not based on samples of Australian honey.

## Introduction

The recent emergence of multiple global threats to the health of honey bees [1, 2], and the increasing illicit international trade in fraudulent or adulterated honey [3, 4] together have stimulated an increasing emphasis on the use of quantitative analyses to demonstrate the geographic and botanical origins of honeys [5]. Melissopalynology, or the pollen analysis of honey, is widely used to verify the claimed geographic and floral origin of honey samples [6–8], and is routinely conducted as part of food quality assurance procedures in some regions, especially the European Union [9].

The Australian honey industry produces 20–30 kilotonnes of honey per year [10, 11], estimated to be worth A$101M in 2014–2015, 4,600 tonnes of which is exported. However, pollen analysis has never been systematically explored as a potential way to verify the floral origins of Australian honeys [12]. The few studies of honeybee foraging preferences in Australia [13, 14], have not investigated the pollen content of honey. Moreover, some previous suggested approaches to the detection of adulterated honey in Australia have not even considered the possibility of pollen analyses [15].

In Australia, the European honey bee (*Apis mellifera*) is an introduced species which is managed by beekeepers and is also widely naturalised [16]. The majority of commercial honey is produced from indigenous vegetation [11], mostly in the relatively well-watered continental margins of the southern half of the continent, on a mixture of private and public land [10]. Natural vegetation in this region is mainly forests and woodlands dominated by species of *Eucalyptus* (Myrtaceae), or, in warmer parts of Australia, the closely related genera *Corymbia* and *Angophora*; collectively, these genera are referred to as eucalypts [17]. Southern Australian eucalypts are evergreen, mostly fast-growing, ecologically opportunistic trees adapted to oligotrophic soils, seasonal to episodic drought, and episodic fire [18]. Many eucalypt species reward bird and mammal pollinators with very heavy nectar and/or pollen production [19], which is also accessible and attractive to honey bees [16]. In the many tropical to subtropical regions outside of Australia where eucalypt species have been planted, and often become naturalised, they frequently have become important, or even dominant, nectar sources for beekeeping in those countries, for example in southern Asia [20], in South America [21–23], in Africa [24, 25] and in other tropical regions [26, 27], and in many countries within the Mediterranean Basin [28–30].

As an analytical technique for food quality assurance, melissopalynology is more intensively applied in Europe than elsewhere. Because of this, published data about the palynological composition of *Eucalyptus* honeys is largely based on analyses of honeys produced within the Mediterranean Basin. Thus it is perhaps not surprising that a pollen analytical study commissioned by Australia’s Rural Industries Research and Development Corporation [12], and carried out by a prominent European food testing consultancy, found that of 20 Australian honey samples described by beekeepers as unifloral *Eucalyptus* or *Corymbia* honeys (that is, in theory, honeys produced primarily from the nectar of a single species [31]), seven were not accepted as unifloral *Eucalyptus* honeys. Of these seven, four were considered primarily “blossom” honeys or honeydew honeys, and one was not accepted as *Eucalyptus* honey at all. The reason for this scepticism by European melissopalynologists was presumably that pollen they identified as *Eucalyptus* did not constitute a sufficiently high proportion of the total pollen in these honeys. International Honey Commission criteria for accepting *Eucalyptus* unifloral honeys are based on examination of 208 European-Mediterranean honey accessions. They stipulate that *Eucalyptus* pollen should on average represent 95%, and a minimum of 83%, of the pollen in a unifloral *Eucalyptus* honey [32]; yet in 13 of RIRDC’s 20 Australian unifloral *Eucalyptus* honey samples, *Eucalyptus* constituted less than 80% of the pollen observed and, in two samples, less than 50%. Assuming that the beekeepers from which these samples were obtained were acting in good faith, this implies that honeys produced in Australia predominantly from natural stands of eucalypts have a substantially different palynological profile from those produced from planted or naturalised eucalypt species growing in the Mediterranean region. One likely explanation for this difference is that eucalypt honeys produced in the Mediterranean region are derived from very few species [30]. By comparison, in Australia there are ~800 species of *Eucalyptus*, dozens of which may be used by beekeepers. This contrast suggests that palynological criteria developed in the Mediterranean for recognising *Eucalyptus* honeys may not provide an adequate basis for evaluating the origin or authenticity of Australian honeys, even if the majority of Australian honeys are predominantly produced from *Eucalyptus*.

In order to develop an understanding of their pollen composition, in this study we examine a range of Australian honeys sourced directly from beekeepers via two major Australian honey packing companies. Although some of the honeys studied here were described by beekeepers as unifloral, the focus of this study is not the authentication of purported unifloral honeys, primarily because species- and even genus-level identification of eucalypt pollen, like that of Myrtaceae generally, is often difficult. Recent studies focusing on both modern and fossil material have confirmed that identification of unknown Myrtaceae pollen grains at species- and genus-level is not often possible without *a priori* knowledge of a (small) number of possible source taxa [33–35]. In the absence of this knowledge, dispersed Myrtaceae pollen may not be reliably identifiable at lower taxonomic level than the tribe [36]. In theory, it should be possible to identify confidently a suite of Myrtaceae pollen observed in an individual honey sample, if the source vegetation for the honey is well known. In this study, however, the source vegetation could not be precisely defined because the geographic localities provided by beekeepers were too generalised to allow this. We therefore focus on the broader issue of whether there are characteristic palynological features that distinguish Australian honeys, as a group, from honeys produced on other continents. We find that the morphological diversity of Myrtaceae pollen is a pervasive feature evident in the majority of Australian honeys. We then address the question whether this characteristic distinguishes Australian honeys from those produced outside of Australia.

## Materials and methods

**Fig 1:**
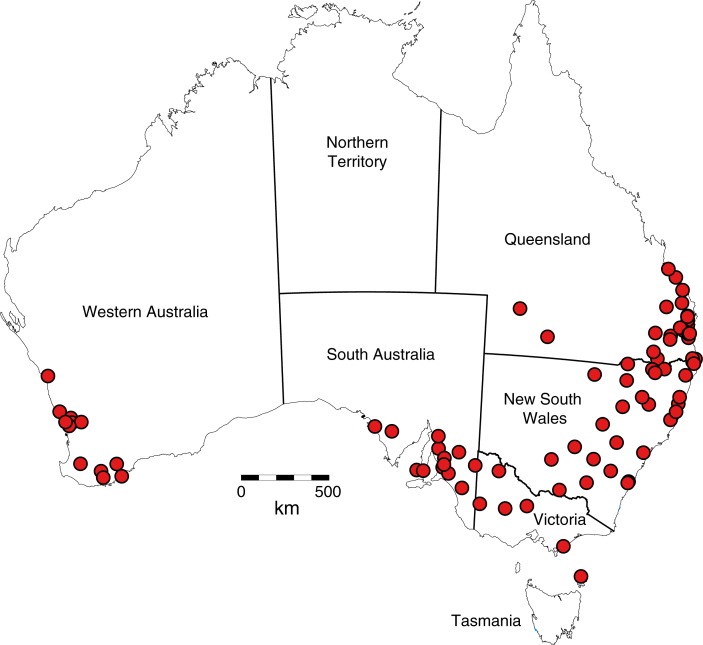
Location of the 173 honey samples within Australia. The data used to produce this figure can be found in the S1 Table.

We processed 173 honey accessions from two major Australian retail honey sellers, Beechworth Honey and Capilano Honey, who supplied approximately 20–40 ml of each honey accession in plastic sample jars. Each sample was taken, by employees of Beechworth and Capilano, from food-grade containers filled with raw honey direct from beekeeper extractions from a single apiary location and beekeeper. Sampling equipment was cleaned by beekeepers between each collection, to ensure no opportunity for contamination. Samples were labelled with a specific tracking identity and shipped via courier to the University of Melbourne where they were analysed in the same year that they were collected. Samples were taken from several regions of Australia (Fig 1), including southern Queensland (mostly in the southeastern corner, but with a small number of samples from the semi-arid southwest of the State), eastern New South Wales, Victoria, southeastern South Australia, and southwestern Western Australia (SW WA). Tasmania was unfortunately excluded, with the exception of one sample from Flinders Island. GPS coordinates for honey collection locations are not available, as beekeepers generally will not divulge this information, for commercial reasons. Spatially generalised locations and corresponding geographic coordinates of honey collection locations, which generally correspond to the nearest town, are provided (S1 Table). For 124 of the samples, extraction dates were available; the majority (75%) of these 124 samples were produced in Spring or early Summer (September through January).

In the laboratory, samples were processed as follows. Approximately 7 ml of warmed honey was taken from each sample jar, using an open-ended, disposable plastic syringe, transferred to a 50 ml, pre-weighed polypropylene centrifuge tube, then weighed to two significant digits on a laboratory balance. Each sample weighed approximately 10 g. Approximately 40 ml of distilled water was added to each sample, and *Lycopodium* tablets, available from Lund University, Sweden, were added to each sample (with 3 ml of 10% HCl to hasten their dissolution), to allow calculation of pollen concentrations [37]. Two different *Lycopodium* tablet production batches were used, containing different numbers of spores; in order to add approximately the same number of spores to all samples, either one or two tablets were used, adding either 20,848 (1 tablet) or 19,332 (2 × 9666) spores, respectively. This ca. 8% difference in number of marker spores added between sample processing batches is small compared to the more than two orders of magnitude difference in pollen concentration between samples (S1 Dataset); it therefore has negligible impact on our pollen concentration calculations [37]. The honey samples were placed into a hot water bath at 80°C until it was possible to homogenise the honey+water mixtures using a vortex mixer. The homogenised dilute honey samples were then centrifuged at 3500 rpm (relative centrifugal force = 2355) [38] for 3 minutes, decanted, rinsed and decanted again. After dehydrating the residues in glacial (100%) acetic acid, the samples were centrifuged and decanted, then acetolysed using a 9:1 mixture of acetic anhydride ((CH_3_CO)_2_O) and concentrated sulfuric acid (H_2_SO_4_) [39, 40]. Acetolysis was used to facilitate comparisons of the pollen grains with similarly acetolysed modern pollen reference collections. After twice centrifuging and decanting, the pollen pellets were dehydrated with 100% ethanol, then small quantities of each pellet were mounted in glycerol on permanent glass slides.

Pollen grains were counted on a Zeiss Axioscope A1 compound microscope at 300×, 600×, and 1500× magnification, with EC Plan-Neofuluar objectives and 16× eyepieces. Pollen was identified by reference to the author’s modern pollen reference collection, published manuals [41–45], published studies of individual families, e.g. [33, 46, 47], and the Australasian Pollen and Spore Atlas (available at apsa.anu.edu.au). Pollen was counted on transects until a pollen sum of 500–600 was achieved. All taxa interpreted as primarily animal-pollinated were included in the pollen sum; taxa interpreted as primarily wind pollinated were excluded from the sum and are not discussed here. Pollen concentrations were calculated using the following formula:

$$\frac{\mathrm{Lycopodium}\;\mathrm{added}}{\mathrm{Lycopodium}\;\mathrm{counted}}\times \frac{\mathrm{pollen}\;\mathrm{counted}}{\mathrm{sample}\;\mathrm{mass}\;(g)}$$ (1)

To reduce the variance in the pollen dataset to a lower number of dimensions, we performed principal component analyses (PCA), and cluster analyses, using Primer 6.1.13 [48]. Analyses were performed on all pollen types achieving at least 1% of the pollen sum in one or more samples, after square root transformation to reduce the influence of dominant pollen types. Cluster analyses were performed on a Bray-Curtis similarity matrix of the data [49], using complete linkage. Histograms and correlations were calculated in Igor Pro 7 [50].

## Results and discussion

### Pollen content of Australian honeys

**Fig 2:**
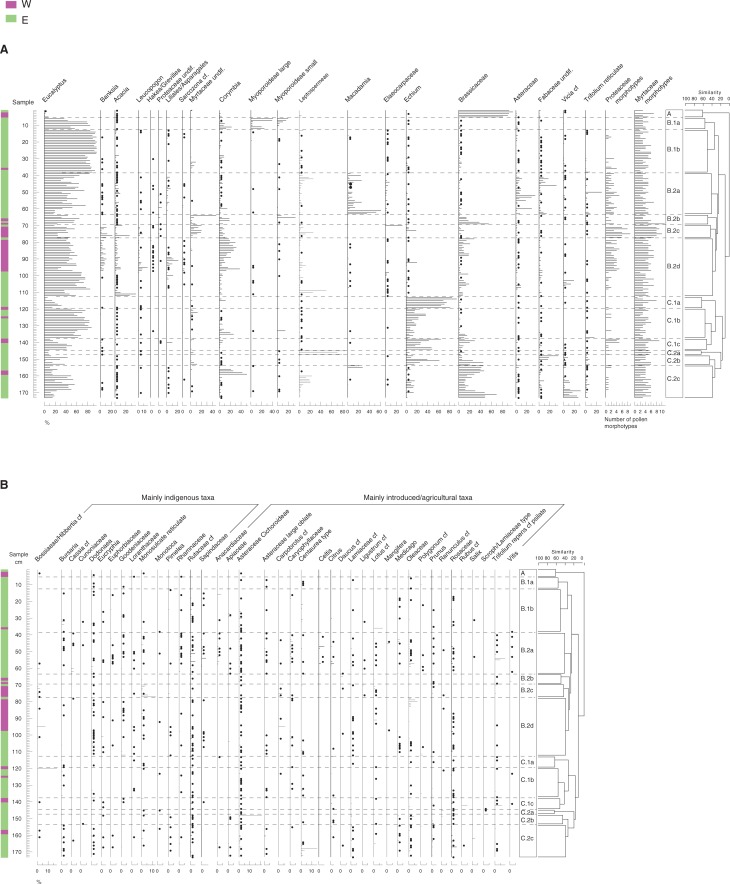
Percentage pollen diagram illustrating the pollen content of the honey samples. Samples are arranged in a sequence determined by cluster analysis. Coloured bar indicates whether each sample was produced in eastern (purple) or southwestern (green) regions of Australia, though note that region was not used as a criterion for the cluster analysis; **a,** the numerically most important taxa; **b**, minor taxa, arranged according to whether probable source species are primarily indigenous to Australia or primarily introduced/agricultural species.

**Fig 3:**
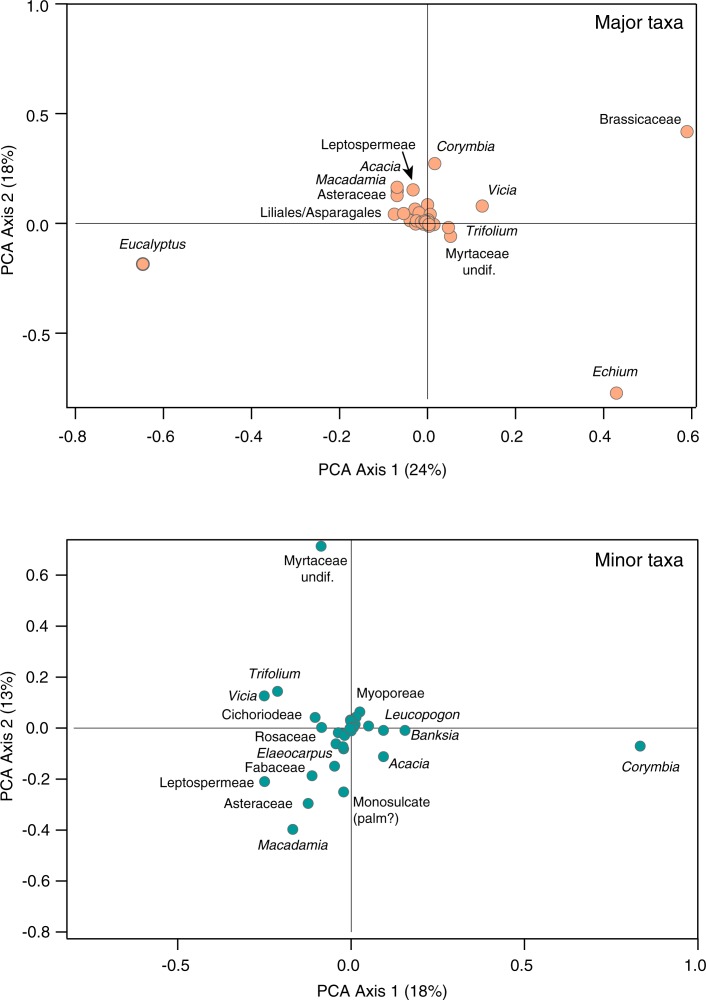
Principal component biplots of pollen types. Upper panel, numerically dominant types, 42% of variance explained by the first two axes; lower panel, numerically minor types, 31% of variance explained by the first two axes.

The pollen assemblages of the 173 honey samples are presented as a pollen percentage diagram (Fig 2A and 2B), arranged in a sequence determined by the cluster analysis. The cluster analysis produces a hierarchical classification of the samples, shown at the right of Fig 2, providing a basis for discussing groups of compositionally similar samples. This analysis, and the distribution of pollen types along the first two Principal Component axes (Fig 3), show that the majority of honey accessions are palynologically dominated by *Eucalyptus* and/or by Brassicaceae (presumably mostly Canola, the widely grown cultivar of rapeseed *Brassica* species, but probably also naturalised *Brassica* species), *Echium* (presumably the introduced and naturalised annual or biennial herb *E*. *plantagineum*, Boraginaceae), *Corymbia*/*Angophora*, Leptospermeae, or *Macadamia*.

**Fig 4:**
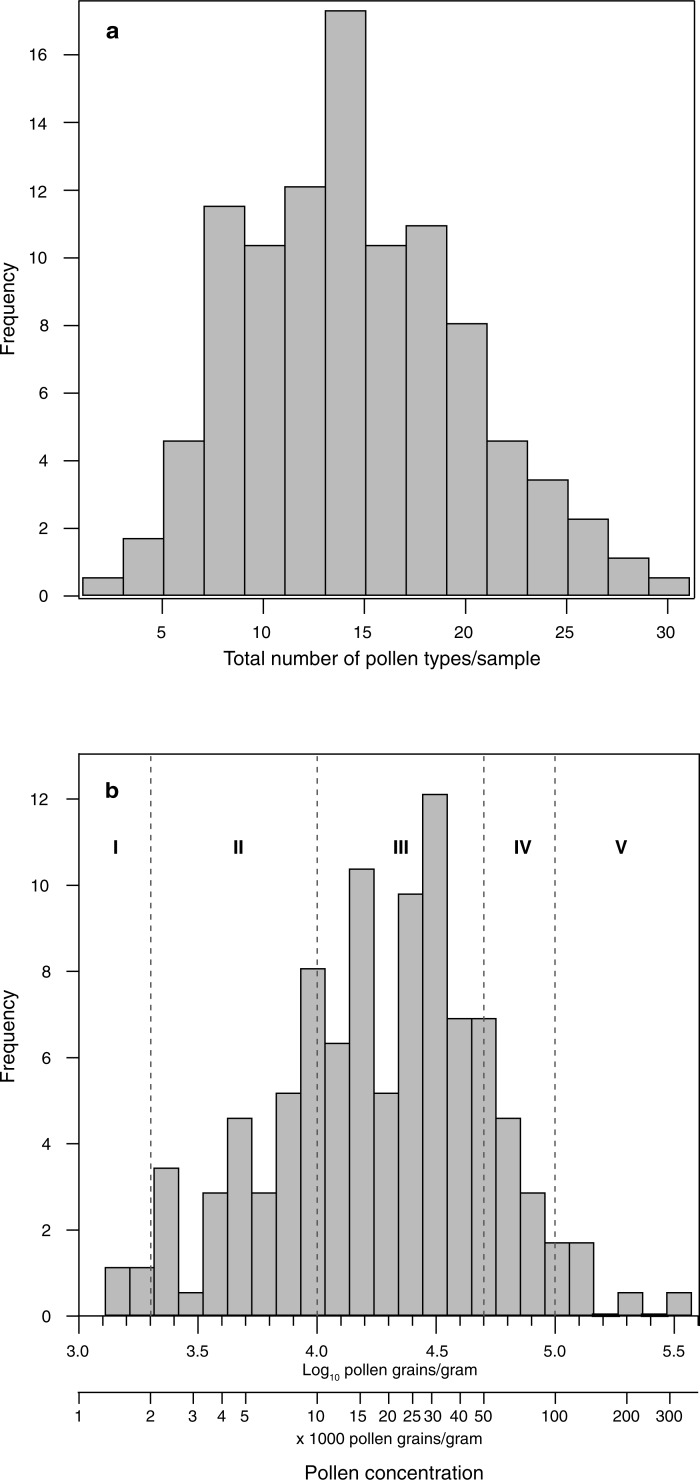
Numbers of pollen types and pollen concentrations. **a**, Histograms of total number of pollen types per sample, and **b**, pollen concentration, on a log_10_ scale, with Maurizio’s groups I-V [7] delineated.

We present the dataset (Fig 2A and 2B) in terms of 61 pollen types. The number of these pollen types observed per sample ranged from two to 31, with an average of 15 (Fig 4A). Pollen concentrations ranged from 1130 to 327,000 pollen grains/g (Fig 4B). Here we describe pollen concentrations in pollen grains/g in order to be consistent with concentrations reported in many other fields, though we note that in the melissopalynology literature pollen concentrations are often reported per 10 g [7]. Only six samples belong in Maurizio’s group I [7], with ≤2,000 pollen grains/g; 47 samples (27% of the dataset) belong to group II, with 2,000–10,000 pollen grains/g; 99 samples (53% of the dataset) belong to group III, with 10,000–50,000 pollen grains/g; 16 samples (9% of the dataset) belong to group IV, with 50,000–100,000 pollen grains/g; and five samples belong to group V, with >100,000 pollen grains/g. We note that the five samples with concentrations >100,000/g were calculated based on observations of very few *Lycopodium* spores in counts of ~600 pollen grains, and therefore should be viewed with caution, though clearly the pollen concentrations in these samples are very high.

The 61 pollen types enumerated in Fig 2 conceal a greater palynological diversity that we could not identify to ‘pollen type’ level. Pollen types of uncertain affinity, or which could be identified only at relatively high taxonomic level, were a persistent difficulty for two distinct reasons. First, the honeys represent the Australian naturalised and agricultural flora, which is poorly documented palynologically, and which extends over a geographic area approximately the size of Europe. At the beginning of the study, we hoped to narrow down the identity of uncertain pollen types produced by introduced plant taxa, through examination of plant species occurrence records (available from the Atlas of Living Australia, www.ala.org.au) within narrowly defined regions where individual honey samples had been produced. In most cases this was not feasible, however, because we had only approximate locality details for each honey sample (nominating a nearby town, at best). Second, the indigenous flora of southern, eastern and western Australia is very species-rich, and the majority of species are animal-pollinated and thus potentially attractive to honey bees, but its palynology remains incompletely documented. The number of candidate species that may have produced a given pollen grain identified at, say, genus level, frequently numbers in the dozens to hundreds. Here, too, the inability to narrowly define collection localities mostly prevented us from reducing the number of possible source species through knowing the particular vegetation in which the bees had foraged. For these reasons, single pollen types recorded at genus level within an individual sample may combine pollen from several local species; while, across the data set as a whole, it is quite possible that many tens of species may have contributed to what we identify as an individual pollen type.

**Fig 5:**
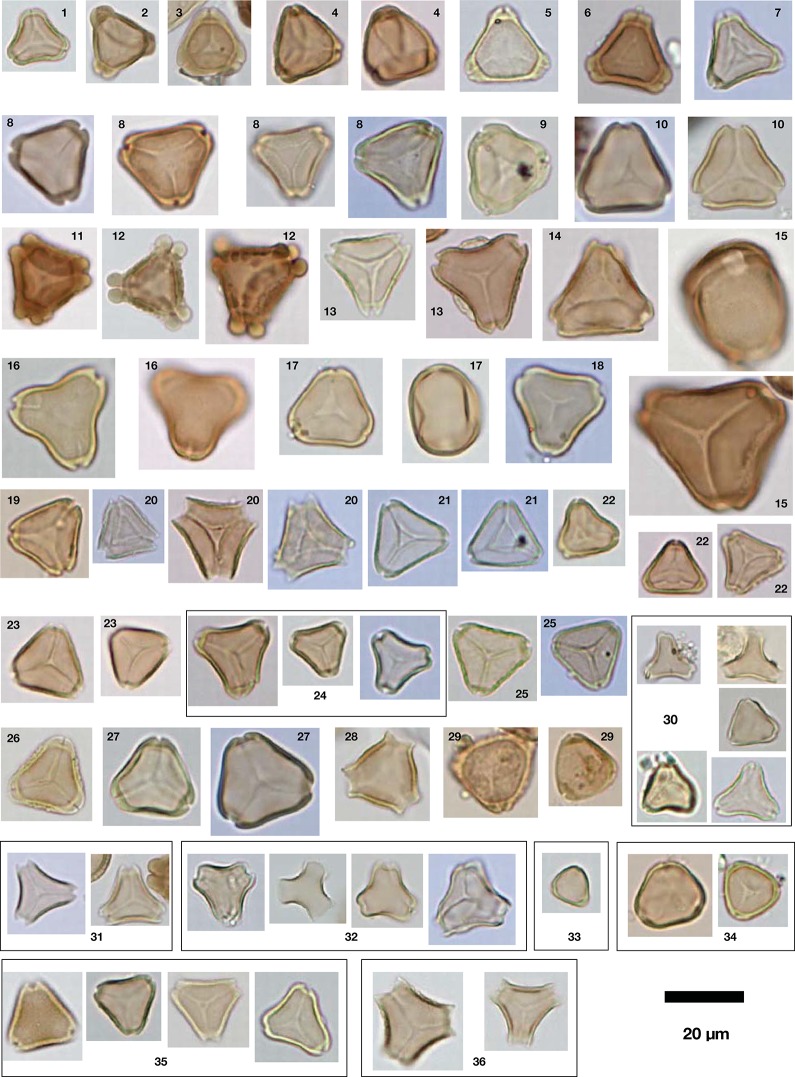
Myrtaceae pollen diversity observed in the honeys. 36 distinct Myrtaceae morphotypes observed in the 173 honey samples, many unassigned below family level. Morphotypes 1–12, parasyncolporate grains with more or less well developed pore thickenings, broadly consistent with *Eucalyptus* species; morphotype 15, large grains, weakly oblate, approaching cubic or spheroidal shape, consistent with some *Corymbia*/*Angophora* species; morphotype 16, with short colpi not reaching the polar region, consistent with some members of the VACDH clade [52]; morphotype 29, regulate grains possibly consistent with tribe Myrteae; morphotypes 30–32 and 34–35, consistent with tribes Leptospermeae and Chamelaucieae; morphotype 33, very small grain possibly consistent with *Tristania*. For brief descriptions, see S1 Appendix.

**Fig 6:**
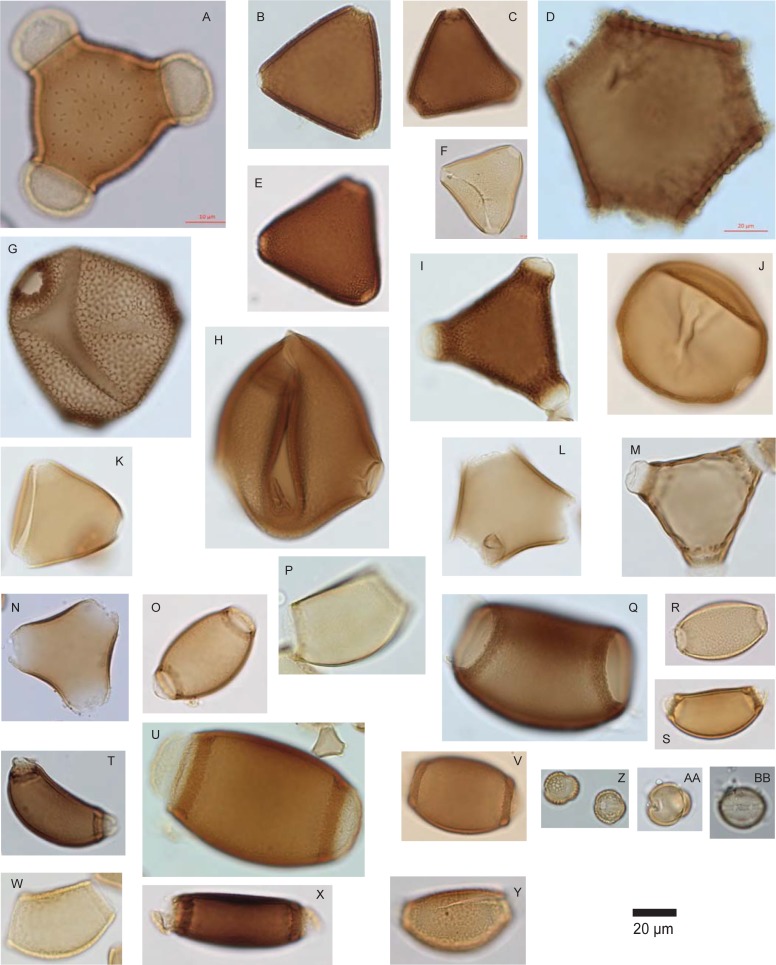
**a-y**, **Pollen types observed in the honeys**. **a-y**, a representative sample of Proteaceae morphotypes observed in this study; **a-n**, triporate Proteaceae, the probable plesiomorphic condition for the family [53]; **o-y**, biporate grains consistent with *Banksia*. **z**, small, spheroidal tricolporate grains with reticulate, heterobrochate exine, lumina becoming smaller near the colpi, consistent with *Bursaria* (Pittosporaceae); **aa-bb**, spheroidal, finely reticulate diploporate grains (ectocolpi have paired endoapertures), consistent with Myoporeae (in Australia, *Myroporum* and *Eremophila*), Scrophulariaceae.

An inability to assign individual names to the observed diversity of pollen grains was especially acute for the ecologically and floristically important families Myrtaceae (Fig 5) and Proteaceae (Fig 6). Both of these families contain large numbers of genera and species in Australia, with ~75 genera and ~1500 species in Myrtaceae, and 45 genera and ~900 species in Proteaceae (Flora of Victoria: https://vicflora.rbg.vic.gov.au/). Although the pollen morphology of these species is moderately well documented, subtle inter-specific pollen morphological variation in both families, most of which is not phylogenetically informative, has long impeded efforts to refine the identification of Myrtaceae and Proteaceae pollen, where they appear in fossil assemblages [36, 51–53]. Several attempts to identify fossil Myrtaceae pollen grains to species level have yielded ambiguous results. For example, Thornhill [35] re-analysed a sedimentary fossil pollen record extending from the present back to ~12,000 years ago, from Bega Swamp, in southern New South Wales. Hope [54] had previously identified 35 distinct Myrtaceae pollen types from these sediments, but Thornhill, comparing the fossil pollen to modern pollen of all Myrtaceae species that now grow near the site, and found that few of Hope’s fossil *Eucalyptus* morphotypes could be consistently assigned to the local species. This example illustrates the difficulty of identifying unknown Myrtaceae types at species level, even for times in the geologically very recent past when environments were similar to today, and even where the modern Myrtaceae flora includes only a few dozen species within a handful of related genera. Identification of unknown Proteaceae pollen to genus or species level presents similar problems [53, 55, 56].

For Myrtaceae and for Proteaceae, we therefore scored pollen grains mostly at family level, with a few identifications at lower taxonomic level. In the Myrtaceae, these include *Eucalyptus* (containing hundreds of species, some of which are difficult to distinguish from some species of *Melaleuca*, also a diverse and ecologically important genus of ~300 species, many of which are very attractive to honey bees); *Corymbia*/*Angophora*, two palynologically indistinguishable sister genera within the tribe Eucalypteae [57]; and the species- and genus-rich tribes Leptospermeae (8 genera and ~160 species in southern Australia) and Chamelaucieae (~27 genera and ~520 species in southern Australia) [58], which probably cannot safely be distinguished from one another palynologically [46]. In addition, a small number of *Eucalyptus* species produce morphologically distinctive pollen, e.g. the gemmate/clavate pollen of *E*. *spathulata* [59]. For typically triporate Proteaceae, grains identified below family level included *Grevillea*/*Hakea* [56], with very heavily thickened exine, though it is likely that assignment of this ‘type’ to *Grevillea*/*Hakea* involves both false positives and false negatives [53]; biporate *Banksia* [60–61], which embraces a broad range of poorly documented intrageneric pollen morphological variability [60, 62]; and the domesticated nut crop *Macadamia*; our confidence in identifying pollen of the latter at genus level relied on the observation that most honey samples described by beekeepers as *Macadamia* were in fact usually dominated by a single Proteaceae type that was morphologically consistent with modern pollen reference preparations of this genus, but *Macadamia* is not highly distinctive morphologically, and is farmed in subtropical regions in which many other indigenous and cultivated Proteaceae occur.

**Fig 7:**
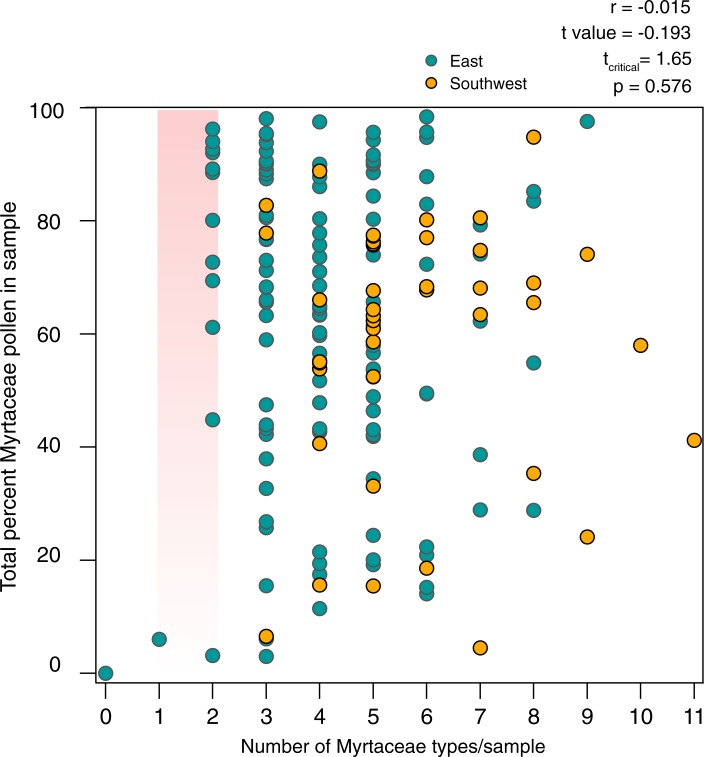
Myrtaceae morphotype diversity and total percent Myrtaceae pollen. Linear correlation between number of Myrtaceae morphotypes per honey sample, and percent Myrtaceae pollen (the sum of *Eucalyptus*, *Corymbia*/*Angophora*, Leptospermeae and Myrtaceae undif.) within the sample, is low and statistically insignificant (*n* = 173, r = -0.015. T-value of -0.193 is less than a critical threshold t_critical_ = 1.65, testing the hypothesis that the correlation coefficient equals zero. Pink bar indicates approximate region in which Mediterranean-region *Eucalyptus* honeys would plot, with gradient indicating that *Eucalyptus* pollen % in Mediterranean honeys can vary from 0 to ~100%, but consistently with only 1–2 Myrtaceae pollen morphotypes. Comparatively few Australian honeys plot in this region.

In an attempt to document the diversity of these two families despite the difficulties described above, we counted the *number* of distinct Myrtaceeae and Proteaceae morphotypes in each sample. Some of these morphotypes, particularly for Myrtaceae, were observed by scanning microscope slides after the pollen sum had been reached, and those morphotypes may constitute as little as ~1 grain per thousand in the pollen assemblage. There is no linear relationship between number of Myrtaceae morphotypes in a sample, and the total percent pollen count of all Myrtaceae in that sample (Fig 7). However, the two samples containing 0 or 1 morphotypes also had very little total Myrtaceae pollen, and of the seven samples with ≤6% Myrtaceae pollen, six have ≤3 Myrtaceae morphotypes. The other 167 samples can exhibit almost any combination of Myrtaceae morphotype diversity and total percent Myrtaceae pollen (from 11.5 to 98.4%).

Thus, a distinctive feature of most of the honey samples is not only, or even primarily, that their pollen assemblages are dominated by *Eucalyptus* and other Myrtaceae, as we might expect, but that many include several (ranging from 0 to 11, mean = 4.6) distinct Myrtaceae pollen morphotypes. In addition, there are a small number of distinctive pollen types produced by endemic Australian taxa, that individually or in combination may help characterise the Australian geographic origin of some honeys. These include *Banksia* (Proteaceae, Fig 6O–6Y), a component of the pollen sum of 35% of all samples; *Bursaria* (Pittosporaceae, Fig 6Z), observed within the pollen sum of 24% of samples; the diploporate Myoporeae (Scrophulariaceae, Fig 6AA–6BB), found mainly in honeys produced in semi-arid southwestern Queensland; and a suite of pollen types observed mostly or exclusively in SW WA samples, including very large echinate *Hakea* (Proteaceae), *Leucopogon* (Ericaceae), and *Sarcozona* cf. (Aizoaceae). However, across the dataset, high Myrtaceae morphotype diversity is the most widespread feature that seems to have potential to uniquely characterise Australian honeys. We enumerated Myrtaceae morphotype diversity only at the scale of individual samples, and made no attempt to identify the same morphotypes in successive honey samples. However, sorting of microphotographs indicated that 36 distinct morphotypes are present in the dataset as a whole (Fig 5). Morphological differences between many of these morphotypes are subtle, and probably would not allow separation of all 36 types if they were encountered within a single sample. However, the majority of the ‘morphotypes’ enumerated in Fig 5 in fact represent several pollen grains that varied along morphological continua (some of this variability is illustrated by documenting more than one grain per morphotype), which may imply that some morphotypes represent more than one species, or more than one genus. Given that the honeys examined here were sourced from a continent-wide diversity of habitats and bioregions [63], it would not be surprising if honey bees had gathered pollen from at least 36 different Myrtaceae species. Therefore, we consider our estimate of 36 Myrtaceae morphotypes to be a conservative characterisation of the morphotype diversity of Myrtaceae in Australian honeys.

**Fig 8:**
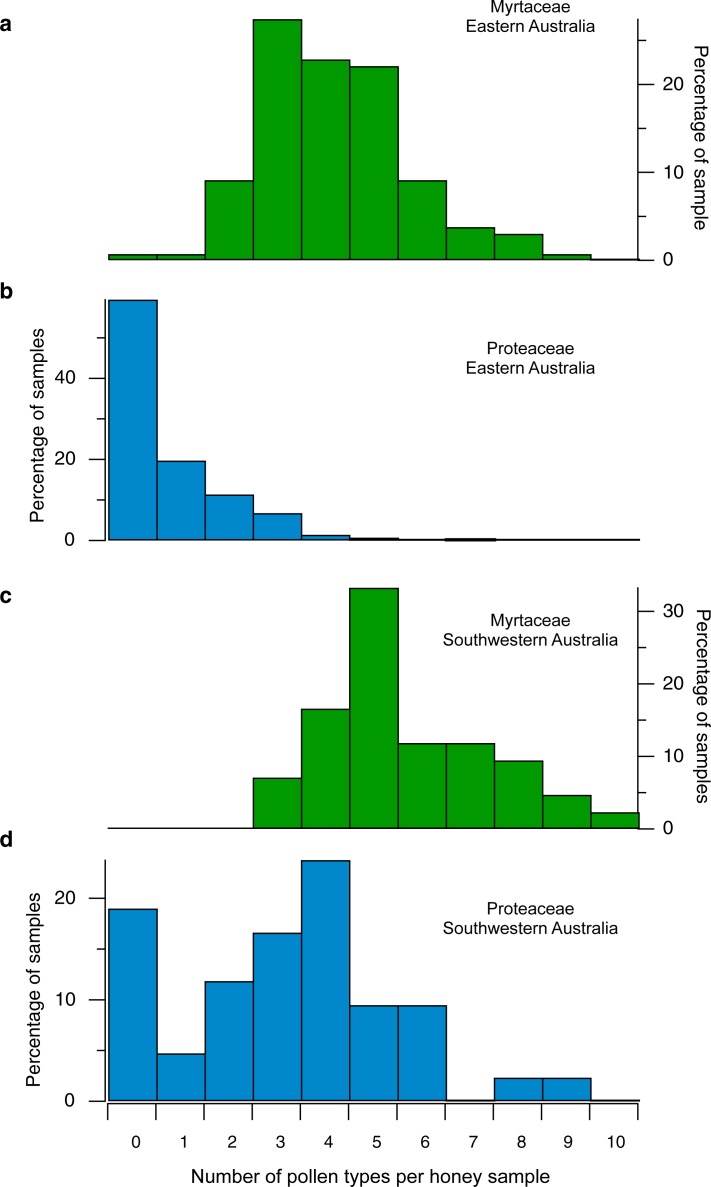
East-west comparison of Myrtaceae and Proteaceae morphotypes diversity. Histograms of number of Myrtaceae (green) and Proteaceae (blue) pollen morphotypes, for eastern (**a,b**) and southwestern (**c,d**) Australian honey samples.

The number of Myrtaceae morphotypes is generally higher in samples from SW WA (mean = 5.8) than from eastern Australia (mean = 4.2) (Figs 7 and 8). For the dataset as a whole, 92% of samples have ≥3 morphotypes; 69% have ≥4, and 48% have ≥5 types. However, a number of samples from SW WA also have high numbers (≥4) of Proteaceae morphotypes, higher than any eastern Australia sample (group B.2c and some samples in group C.1c of Fig 2; Fig 8). The combined high Myrtaceae and Proteaceae morphotype diversity in these samples may reflect the fact that the SW WA floristic region [64] supports a larger number of species in both Proteaceae (~1000 taxa recognised at and below species level, in 15 genera), and in Myrtaceae (~1500 taxa recognized at and below species level, in 47 genera), (FloraBase, the Western Australian Flora, consulted 20 Jan 2018: https://florabase.dpaw.wa.gov.au), with correspondingly greater diversity at the hectare to km^2^ spatial scales relevant to honey bee foraging distances, than corresponding vegetation in eastern Australia [65, 66]. Alternatively, or in addition, the important feature of the southwest may be that species-rich shrublands dominated by Proteaceae and Myrtaceae, providing diverse floral sources throughout much of the year, are more widespread and extensive in the SW WA floristic region than in most regions of the eastern States [67, 68].

In contrast to honeys gathered largely in indigenous vegetation, it seems at first that it should to be difficult to authenticate the Australian origin of honey samples produced in predominantly agricultural landscapes, where bees had access mainly to introduced floral resources. Examples include Canola honeys, which were found to be dominated by Brassicaceae pollen, represented by groups A, B2b and C.2c (Fig 2A); and honeys dominated by the pollen of *Echium* (largely *E*. *plantagineum*, an extremely heavy pollen producer [69], known by Australian apiarists as ‘Salvation Jane’), represented by groups C (Fig 2A). Nevertheless, some of these honeys include large amounts of pollen from indigenous taxa, or at least contain highly diagnostic indigenous taxa. For example, group C.1c (Fig 2A), consisting of seven samples with medium to high *Echium* pollen percentages, combines three samples from SW WA with four from eastern Australia. The SW WA samples each have 4 to 5 Proteaceae morphotypes, and 5 to 9 Myrtaceae morphotypes, despite the fact that *Echium* constitutes ≥60% of the sum in one of the samples, and none have more than ~25% *Eucalyptus* pollen. Their Proteaceae and Myrtaceae content makes these samples clearly diagnostic of a SW WA origin, despite apparently having been produced in a mosaic of agricultural land and indigenous vegetation, and despite unpromising beekeeper descriptions of them, as mixtures of “WA wildflower” or “WA mix honey” and “Salvation Jane”. A still more striking example is provided by sample 69 (Fig 2A, group B.2b), which has the highest recorded number of Myrtaceae morphotypes, 11 (though possibly more; 13 images in Fig 5, of morphotypes 4, 8, 11–16, 22–25 and 30, are sourced from this sample), but only modest percent Myrtaceae pollen (Fig 7). The sample was described by the beekeeper, based in Ongerup, SW WA, as a mixture of Canola and Yate (*Eucalyptus cornuta*), which is perhaps consistent with it having 55% Brassicaceae pollen and only 24% *Eucalyptus* pollen. Clearly, not all honeys produced primarily within agricultural landscapes provide bees with such rich indigenous pollen sources, but some of these honeys will nevertheless be as readily identifiable as Australian in origin, as honeys produced entirely from indigenous vegetation.

### Comparison with the Myrtaceae content of honeys from other regions

**Fig 9:**
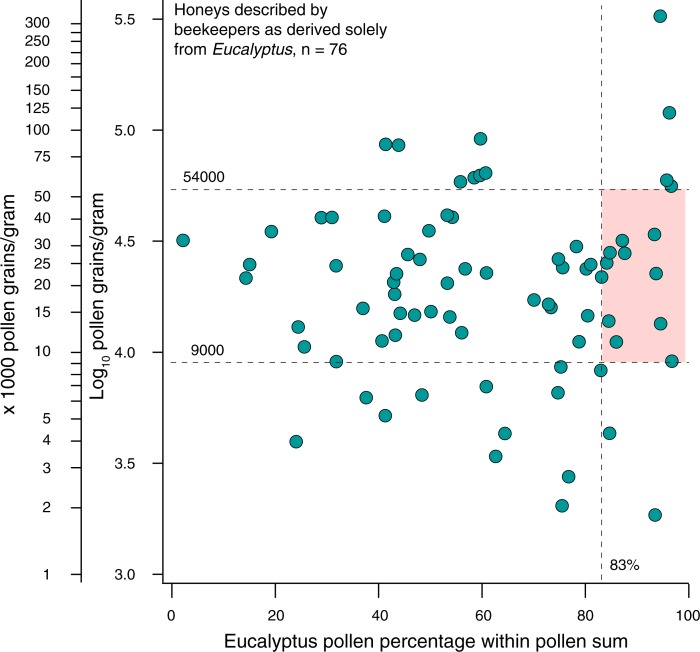
Relationship between percent *Eucalyptus* pollen and total pollen concentration, in purportedly unifloral *Eucalyptus* honeys. Percent *Eucalyptus* pollen in 76 samples described by Australian beekeepers as derived solely from one or more *Eucalyptus* species (that is, unifloral, or in some cases bifloral *Eucalyptus* honeys), vs. total pollen concentration, plotted with a log_10_ scale. For comparison, pink shading indicates the International Honey Commission’s criteria [32], based on percent *Eucalyptus* pollen (>83%) and range of total pollen concentration (9000–54,000 pollen grains/g), for acceptance of a honey as a unifloral *Eucalyptus* honey. Comparatively few Australian *Eucalyptus* unifloral honeys plot in this region.

Does the observed diversity of Myrtaceae pollen morphotypes clearly set Australian honeys apart from those produced on all other landmasses? In particular, is this diversity a distinct enough feature that it can allow differentiation of Australian honeys even from *Eucalyptus* honeys produced in other countries, many of which are considered unifloral honeys strongly dominated by the nominate pollen type [28]? Our answer to this question is tentative, because we were not able to directly examine unifloral *Eucalyptus* honeys produced outside of Australia. However, the majority of *Eucalyptus* honeys produced in the Mediterranean region appear to contain very few Myrtaceae morphotypes (Table 1). This is probably not unexpected, since that region has only a single indigenous Myrtaceae species, *Myrtus communis*, and since *Eucalyptus* honeys there are produced almost exclusively from just two species, *E*. *globulus* and *E*. *camaldulensis* [30, 70], perhaps because these two species are more widespread and abundant than any other *Eucalyptus* species in that region [71]. The production of European *Eucalyptus* honeys from such a narrow sample of the genetic diversity of the genus, and from dense stands of trees that often form monocultures [71, 72], probably explains the very high minimum threshold for percent *Eucalyptus* pollen, and the carefully circumscribed range of pollen concentrations, applied by the International Honey Commission (IHC) for accepting the authenticity of a *Eucalyptus* honey (Fig 9). It is clear, however, that *Eucalyptus* honeys produced in Australia are much more diverse, in both of these parameters.

**Table 1:** Number of Myrtaceae pollen types reported in melissopalynological studies in Mediterranean countries.

Country	No. Myrtaceae types/total pollen types	Myrtaceae taxa recorded	Honeytype/source	No. of samples	Reference
Algeria	4/~40	*Eucalyptus*, *Myrtus*. *Leptospermum*, Myrtaceae	Mixed	11	[73]
Algeria	1/124	*Eucalyptus*	Mixed	66	[88]
Greece	1/142	*Myrtus*	Mixed	674	[89]
Greece	1/229	*Eucalyptus*	Mixed	285	[82]
Greece	2/126	*Eucalyptus*, *Myrtus*	Thyme	135	[83]
Italy	1/≥35	*Eucalyptus*	Mixed	44	[8]
Italy & Spain	1/21	*Eucalyptus*	*Citrus*	31	[90]
Morocco	1/29	*Eucalyptus*	*Eucalyptus*	29	[29]
Portugal	1/23	*Eucalyptus*	*Eucalyptus* unifloral	31	[30]
Portugal	1/21	*Eucalyptus*	Mixed	45	[91]
Portugal	1/46	*Eucalyptus*	*Eucalyptus*	13	[28]
Sardinia	1/70	*Eucalyptus*	*Eucalyptus*	31	[92]
Canary Islands, Spain	1/62	*Eucalyptus*	Mixed	24	[93]
Spain	1/28	*Eucalyptus*	Putatively *Erica* unifloral	11	[94]
Spain	1/144	*Eucalyptus*	Mixed and *Castanea*	599	[95]
Spain	2/10	*Eucalyptus*, *Myrtus*	*Eucalyptus*	19	[70]
Spain	2/47	*Eucalyptus*, *Myrtus*	Mixed	20	[96]
Spain	1/84	*Eucalyptus*	*Eucalyptus*	40	[28]

Mixed = mixture of unifloral and polyfloral honeys

The IHC’s narrow circumscription of what constitutes a *Eucalyptus* honey is consistent with the Mediterranean melissopalynology literature. For example, Ouchemoukh et al. [73] recorded four Myrtaceae types in 11 Algerian honey samples, but no individual sample contained more than two Myrtaceae types. Rodríguez et al. [70] attempted to distinguish the pollen of *Myrtus communis* from *Eucalyptus*, in honeys from southern Spain produced in vegetation containing both genera. They explicitly noted that the honeys, with Myrtaceae pollen ranging from 83–97% “did not reveal any great pollen type diversity” (1548). They were able to distinguish *M*. *communis* from *E*. *camaldulensis* pollen, though they used only the subtle difference in the size of the grains (14–17 μm vs. 19–20 μm, respectively). In summary, Mediterranean honeys generally contain no more than 2 Myrtaceae morphotypes (Table 1), despite some honeys having very high *Eucalyptus* values; in this feature, they strikingly differ from most of the 173 Australian honeys (Fig 7). This comparison suggests that the presence of as few as 3 or, perhaps more conservatively, 4 Myrtaceae morphotypes would unambiguously distinguish an Australian honey from *Eucalyptus*-dominated honeys produced in the Mediterranean region. However, what about honeys from regions, such as South America, with rich indigenous Myrtaceae biodiversity?

South America supports a very large number of Myrtaceae species and genera. The family represents more than 10% of all tree species in eastern Brazilian Atlantic forests [74, 75], and there are >1000 species in 23 genera in Brazil alone (Floro do Brasil, (http://floradobrasil.jbrj.gov.br/reflora/floradobrasil/FB171). Despite this diversity, South American honeys do not seem to exhibit a high diversity of Myrtaceae pollen types (Table 2). Sanchez et al. [76] refer to two types of Myrtaceae honey, one from *Blepharocalyx*, another from “native Myrtaceae in forests, where the diversity of this family is greater”. [77] wrote “in the Andean region, pollen from …*Luma*…*Myrceugenia*… and some species of *Eucalyptus*…were classified as Myrtaceae” [that is, at the undifferentiated family level](149). Yet Myrtaceae, and several introduced *Eucalyptus* species, may be the taxa most frequently visited by *A*. *mellifera* and several other indigenous bee species in Brazil, on the outskirts of Sao Paulo [78]. Pollen foraging studies identified a range of eusocial bee species visiting multiple Myrtaceae species including several *Eucalyptus* species [78, 79] (Table 2), but this eclecticism does not seem to be borne out, or cannot be identified, in pollen identifications of actual honey. Barth [21]wrote, “Myrtaceous pollen grains, except *Eucalyptus*, are summarized in the *Myrcia* pollen type…while the numerous species cannot be separated by their pollen morphology” (90). The only exception we could locate was a study [80] that recorded 5 Myrtaceae pollen types (two *Myrcia* types, one *Eugenia* type, one *Eucalyptus* type, and one undifferentiated Myrtaceae type) out of 114 types in total from 27 honey samples. It is unclear how many Myrtaceae types were present in individual samples, but since *Myrcia* was observed in 94% of samples, *Eucalyptus* in 41%, and *Eugenia* in 18%, almost certainly some of the samples must have contained two, and perhaps a few contained three Myrtaceae types.

**Table 2:** Number of Myrtaceae pollen types reported in melissopalynological and pollen foraging studies in South America.

Country	No. Myrtaceae types/total pollen types	Myrtaceae taxa recorded	Honeytype/source	No. of samples	Reference
Argentina	2/139	*Eucalyptus*, Myrtaceae undif.	Mixed	140	[77]
Argentina	1/88	Myrtaceae undif.	Mixed	58	[97]
Argentina	3/109	*Eucalyptus*, *Blepharocalyx*, Myrtaceae undif.	Mixed	157	[76]
Argentina	2/119	*Eucalyptus*, *Myrcianthes*	Mixed	38	[98]
Argentina	2/86	*Eucalyptus*, *Myrcianthes*	Mixed & unifloral (not *Eucalyptus*)	33	[99]
Argentina	1/71	*Eucalyptus*	Unifloral (not *Eucalyptus*)	59	[100]
Argentina	2/120	*Eucalyptus*, *Eugenia*	Mixed	189	[81]
Argentina	1/63	*Eucalyptus*	Mixed	30	[101]
Argentina	1/109	*Eucalyptus*	Mixed	65	[102]
Brazil	2/≥30	*Eucalyptus*, *Myrcia*	Propolis	11	[103]
Brazil, Rio de Janeiro (Atlantic Forest)	2/33	*Eucalyptus*, Myrtaceae undif.	Mixed & unifloral (including *Eucalyptus*)	11	[104]
Brazil, São Paulo (Atlantic Forest)	5/114	*Eucalyptus*, *Eugenia*, *Myrcia*, 2× Myrtaceae undif.	Mixed	34	[80]
Brazil, Rio de Janeiro (mangroves)	4/32	*Eucalyptus*, *Eugenia*, *Jambosa*, *Myrcia*	Mixed	NA	[105]
Chile	2/66	*Eucalyptus*, *Luma*	Mixed	13	[106]
Uruguay	1/66	*Eucalyptus*	Mixed	21	[22]
Brazil	Site 1: 5/39	*Eucalyptus*, *Eugenia*, *Myrcia*, *Psidium*, Myrtaceae undif.;	*A*. *mellifera* pollen foraging study	69 from two sites	[107]
Brazil	Site 2: 6/35	*Eucalyptus*, *Eugenia*, *Myrcia*, 3× Myrtaceae undif	*A*. *mellifera* pollen foraging study	69 from two sites	[107]
Brazil, Pará (Amazon)	2/23	*Myrcia*, *Syzygium*	*Tetragonisca angustula* pollen foraging study	103	[108]
Brazil, Pará (Amazon)	3/32	*Eugenia*, *Myrcia*, *Psidium*	*T*. *angustula* pollen foraging study	23	[109]
Brazil, Rio de Janeiro (Atlantic Forest)	2/7	*Eucalyptus*, *Myrcia*	*Melipona quadrifasciata* pollen foraging study	13	[110]
Brazil, Rio de Janeiro (Atlantic Forest)	2/39	*Eucalyptus*, *Myrcia*	*T*. *angustula* pollen foraging study	NA	[111]
Brazil, São Paulo (Atlantic Forest)	1/68	*Eucalyptus*	*A*. *mellifera* pollen foraging study	5 hives	[86]
Brazil, Sergipe	3/46	*Myrcia*, *Psidium*, *Myrtaceae* undif.	*A*. *mellifera* pollen foraging study	12	[112]
Colombia (Andes)	3/126	*Eucalyptus*, *Myrcia*, Myrtaceae undif.	*A*. *mellifera* pollen foraging study	86	[113]

Mixed = mixture of unifloral and polyfloral honeys

Why should South American honeys generally include few discernible Myrtaceae pollen types, relative to Australia, when South America supports such a large Myrtaceae flora? First, it is worth considering whether we used different criteria to distinguish Myrtaceae ‘morphotypes’ than the criteria typically used by melissopalynologists to distinguish pollen types, either in South America or, for that matter, in Mediterranean countries. However, there is abundant evidence that South American and Mediterranean melissopalynologists routinely make very subtle pollen morphological distinctions. For example, in South American honeys, Benitez-Bosco et al. [80] recorded 8 named Asteraceae and 20 Fabaceae types; Salgado-Laurenti et al. [81] recorded 30 named Fabaceae types; Sanchez et al. [76] recorded 14 Asteraceae types and 16 in Fabaceae. In Greek honeys, Dimou et al. [82] recorded 16 named types in Asteraceae, 15 in Fabaceae, and 12 in Rosaceae, including 4 *Prunus* types; Karabournioti et al. [83] recorded 24 types in Asteraceae, 18 in Lamiaceae. Thus, it seems unlikely that discernible pollen morphological variability in Myrtaceae has been overlooked by melissopalynologists.

Second, all but one South American Myrtaceae species belong to a single tribe, the fleshy-fruited tribe Myrteae [58], the most generically rich tribe in the family (2500 species in 49 genera) [75, 84]. Australia has ~75 species in 11 genera in the Myrteae [58, 84], mostly confined to tropical and subtropical rainforests, a biome which appears to be relatively little used by Australian honey bees, and does not seem to be represented well in our honey samples. Only one of our morphotypes is possibly consistent with the pollen of Myrteae (Fig 5, type 29). Thornhill [35] suggested that Myrtaceae fossil pollen types probably conservatively represent tribes, sometimes genera. However, although there is a large difference in tribal diversity between the South American and Australian Myrtaceae floras, with most southern Australian Myrtaceae species belong to one of five tribes (Eucalypteae, Melaleuceae, Syzygieae, Leptospermeae, and Chamelaucieae), these five tribes alone cannot explain the larger difference in Myrtaceae morphotype diversity between South America and Australia. Apparently, most of these five species-rich, Australia-centred Myrtaceae tribes simply encompass a greater pollen morphological diversity than is expressed within Myrteae, despite the great generic and species-level diversity of the latter. In addition, the attractiveness of different Myrtaceae species to honey bees appears to be highly variable between species and, possibly, between tribes [58]. These differences relate to differences in pollen and nectar production and presentation, among other factors, and may explain the preference of honey bees in South America for foraging pollen of *Eucalyptus* [85], even in the Brazilian Atlantic Forest region [21, 86], where Myrtaceae is a very important part of the forest flora [75].

Myrtaceae morphotype diversity may similarly distinguish Australian Leptospermeae-dominated honeys from Manuka (*Leptospermum*) honeys produced in New Zealand. For example, for the seven Australian honey samples with >25% percent Leptospermeae pollen (Fig 2, but dispersed into several groups), the average number of Myrtaceae morphotypes ranges from 3 to 8, with an average of 5.3. By comparison, pollen analyses of Manuka and other New Zealand honeys [87] indicate that no New Zealand honey includes more than three morphotypes (*Leptospermum*, *Metrosideros*, and *Eucalyptus*).

In summary, for the most part there seems little danger of confusing at least the majority of the Australian honeys with honeys produced in New Zealand, in South America, or in the Mediterranean region. Honeys in any of the latter regions are unlikely to include more than 3, in most cases no more than 2, Myrtaceae pollen morphotypes. Based on these comparisons, and depending on the desired level of confidence, the geographic origin of most of the 173 Australian honey samples could be diagnosed based solely on the number of observed Myrtaceae morphotypes: 69% (120/173) contain ≥4 Myrtaceae pollen morphotypes, while 92% (159/173) contain ≥3 morphotypes, though it would also be useful to pay attention to the likely generic or tribal affinities of each morphotype (Fig 5). This assessment ignores other, non-Myrtaceae pollen types within Australian honeys, though some of these are obviously important and may be equally diagnostic of an Australian origin. But Myrtaceae diversity appears to be the most pervasive characteristic of A. *mellifera* honey in Australia, where honey bees have had access to indigenous vegetation floral resources.

## Conclusions

In this study, we documented the pollen content of 173 *Apis mellifera* honey samples produced in most of southern Australia’s commercial beekeeping regions. We found that the majority of Australian honeys are dominated by *Eucalyptus*. The importance of Myrtaceae in the honeys is unsurprising, since southern Australia’s forest flora is ecologically dominated by *Eucalyptus*, and many *Eucalyptus* species provide floral resources very attractive to honey bees. Other numerically important pollen in some samples includes *Corymbia*/*Angophora*, Leptospermeae and other undifferentiated Myrtaceae; *Acacia*, *Macadamia*, Brassicaceae and *Echium*. Our aim was to examine whether the pollen content of Australian honeys could be used to verify their geographic authenticity, at continental scale, in the context of honeys produced elsewhere in the world. We found that the number of Myrtaceae pollen morphotypes is possibly the most characteristic feature of the honey samples. As a metric for evaluating the authenticity of purported Australian honeys, it would have the benefit that it does not require a highly detailed understanding of the distribution of pollen morphological features within the many genera and species within the Australian Myrtaceae. Additional studies of Australian honey should be conducted, and existing International Honey Commission criteria for authenticating *Eucalyptus* honeys should not be relied upon for Australian honeys, since those criteria are not based on samples of Australian honey.

## Supporting information

S1 Appendix Brief descriptions of Myrtaceae morphotypes featured in Fig 5. (DOCX)

S1 Dataset Pollen percentage data. (TXT)

S1 Table Localities of honey producing sites. (DOCX)
