# Supplementary material for: Pollen analysis of Australian honey
Source: PLoS One. 2018 May 16;13(5):e0197545. doi: 10.1371/journal.pone.0197545 (PMC5955576; doi:10.1371/journal.pone.0197545)
Supplement: S1 Appendix — (DOCX) [file pone.0197545.s001.docx]

**SI Appendix: Brief descriptions of Myrtaceae morphotypes featured in Figure 5**

**1**. Subtle pore thickening, gently concave outline and curving colpi

**2**. Small, pores v thick relative to mesocolpal exine thickness

**3**. Pores v thickened, nearly aspidate, within triangular polar outline

**4**. Parasyncolporate, curving colpi, thickened pores, psilate, gently convex amb with rounded or slightly protruding apices; a generic *Eucalyptus* type

**5**. Exine granulate/scabrate, thin straight colpi, pore thickening great, relative to mesocolpal exine.

**6**. Pores v thickened, near aspidate, protruding in polar view; straight colpi sharply bifurcating to form distinct triangular polar apocolpal region.

**7**. Parasyncolporate, concave amb curvature extending into drawn out points at the apices. Exine psilate, pores pincer like, aspidate.

**8**. Pore thickening largely endoapertural, forming aspidate pore? Otherwise relatively thin exine, with amb sides straight. A small-grained *Corymbia* or *Angophora*?

**9**. Unusual degree of exine thickening, continuous from mesocolpal to pore regions though enhanced further in pore region; exine thickly laminated near pores. Amb sides concave, apices broadly rounded.

**10**. Parasyncolporate, psilate, curving colpi define apocolpal field with concave margins. No pore thickening. Amb outline gently concave, and more or less notched apices.

**11**. Exine thickened throughout, approaching rugulate; almost certainly *Eucalyptus marginata*.

**12**. Pore thickening extended into globular processes, exine more or less gemmate. SW WA samples only, cf. *Eucalyptus spathulata*

**13**. Obscurely parasyncolporate, pore thickenings confined to lateral margins of pore, and/or pore very open. Pores resemble thickened pincers. Exine apparently scabrate in equatorial region.

**14**. Large, with irregular thickness of exine in cross section. Parasyncolporate with straight colpi, exine psilate, no consistent pore thickening.

**15**. Large, syncolporate or with very small apocolpal field often including minute polar island (a region of ektexine fitting within the apocolpal field, [36]); psilate or scabrate, particularly in equatorial mesocolpal region; gently concave amb; grains globose to moderately oblate, in contrast to typical peroblate shape of most Myrtaceae. Consistent with many *Corymbia*/*Angophora* species.

**16**. Brevicolpate, with colpi short and terminating well before reaching polar region; amb distinctly concave, apices bluntly pointed, grain shape effectively three lobed. Consistent with some species within the VACDH clade [36].

**17**. Amb smoothly rounded at apices, slightly concave amb outline, parasyncolporate with variable development of apocolpal field with concave sides. Similar to *Acmena*.

**18**. Demisyncolpate, with colpi confined to polar region, not reaching equator; exine psilate, gently concave amb outline with rounded, notched apices, no pore thickening.

**19**. Little or no pore thickening; parasyncolporate, gently convex amb outline, psilate.

**20**. Pores with no thickening, straight triangular widely notched amb; triangular straight to concave apocolpal field. Undulating optical section implies faintly rugulate equatorial exine, Amb outline ranging from straight to concave. Probably >1 species illustrated here.

**21**. Tightly defined apocolpal field, similar to types 13 and 19, but amb with rounded apices.

**22**. Small, amb outline straight to concave, amb rounded to slightly protruding apices, small apocolpal field with clearly demarcated small polar island.

**23**. Lateral margins of pores with distinct, protruding, unacetolysed, transparent tips, syncolporate or narrowly parasyncolporate, amb and colpi straight to curved.

**24**. Syncolporate, ‘pincer’ like pores forming a vestibulum, exine psilate, slightly to strongly concave amb outline, apices protruding slightly. cf some *Syzygium* species.

**25**. Amb outline straight, exine scabrate throughout, syncolporate with straight colpi. Vestibulum developed in one specimen.

**26**. Pores thickened, syncolporate, colpi straight, amb outline gently concave, exine psilate at poles, but strongly scabrate/rugulate in equatorial region.

**27**. Pores thickened, but syncolporate and therefore inconsistent with most *Eucalyptus* species; exine psilate, amb outline gently convex or concave, with apices rounded. More robust, with thicker exine than type 10.

**28**. Amb apices widely notched and flared because of divergent angle of protruding pore margins. Exine thins at pore margins. Colpi short, leading to large apocolpal field, amb sides convex.

**29**. Rugulate/granulate, gently convex sides, pore protruding: Myrteae cf?

**30**. Very small, faintly scabrate to granulate, syncolporate, colpi very narrow, straight to curving, sides concave, apices in some cases protruding. Cf. Leptospermeae/Chamelaucieae

**31**. Small, colpi straight and very narrow, amb sides distinctly concave, pores slightly thickened at margins. Cf. Leptospermeae/Chamelaucieae

**32**. Small, amb outline strongly concave, amb outline truncate at apices because pore openings very wide relative to grain size, so grain forms a “Y” in polar view. Apparently several species with these features. Cf. Leptospermeae/Chamelaucieae

**33**. Very small (≤10 µm), indistinct colpi and pores.

**34**. Moderately small grains with rounded, faintly convex amb and more or less truncate apices.

**35**. Complex of Leptospermeae/Chamelaucieae cf. types: small, straight sided triangular, syncolporate to weakly parasyncolporate, straight thin colpi, obscurely to prominently notched apices, variably scabrate.

**36**. Similar to type 28 but distinctly syncolporate rather than parasyncolporate, and with straight colpi. Pores thickened only on lateral margin, where transparent, unacetolysed; amb apices forming a wide notch; colpi straight, exine psilate.
