# Supplementary material for: Pollen analysis of Australian honey
Source: PLoS One. 2018 May 16;13(5):e0197545. doi: 10.1371/journal.pone.0197545 (PMC5955576; doi:10.1371/journal.pone.0197545)
Supplement: S1 Table — (DOCX) [file pone.0197545.s003.docx]

**SI Table. Localities of honey producing sites**

| **Locality** | **States** | **Latitude** | **Longitude** |
| --- | --- | --- | --- |
| Albany Creek | Queensland | -27.35 | 152.97 |
| Allora | Queensland | -28.03 | 152.05 |
| Alstonville | New South Wales | -28.84 | 153.45 |
| Batemans Bay | New South Wales | -35.71 | 150.18 |
| Beaudesert | Queensland | -27.99 | 153.00 |
| Beerwah | Queensland | -26.86 | 152.96 |
| Birdwood | South Australia | -34.82 | 138.97 |
| Blackstone | Queensland | -27.63 | 152.8 |
| Bonshaw | New South Wales | -29.21 | 151.37 |
| Boyup Brook | Western Australia | -33.83 | 116.41 |
| Bundaberg | Queensland | -24.87 | 152.35 |
| Caboolture | Queensland | -27.07 | 152.97 |
| Cecil Hills | New South Wales | -33.89 | 150.85 |
| Chiltern | Victoria | -36.15 | 146.60 |
| Clare | South Australia | -33.84 | 138.61 |
| Coolah | New South Wales | -31.84 | 149.68 |
| Coraki | New South Wales | -28.98 | 153.3 |
| Corny Point | South Australia | -34.96 | 137.06 |
| Cranbrook | Western Australia | -34.3 | 117.55 |
| Cunnamulla | Queensland | -28.08 | 145.69 |
| Darlington Point | New South Wales | -34.57 | 146.00 |
| Deepwater | Queensland | -24.43 | 151.95 |
| Dongara | Western Australia | -29.25 | 114.93 |
| Flinders Island | Tasmania | -39.98 | 148.05 |
| Gingin | Western Australia | -31.34 | 115.91 |
| Glass House Mountains | Queensland | -26.9 | 152.95 |
| Glen Innes | New South Wales | -29.75 | 151.74 |
| Grafton | New South Wales | -29.68 | 152.93 |
| Gympie | New South Wales | -26.19 | 152.67 |
| Harden | New South Wales | -34.55 | 148.37 |
| Inverell | New South Wales | -29.76 | 151.11 |
| Ipswich | Queensland | -27.62 | 152.76 |
| Jamestown | South Australia | -33.21 | 138.60 |
| Kamarooka | Victoria | -36.49 | 144.38 |
| Kapunda | South Australia | -34.34 | 138.91 |
| Kempsey | New South Wales | -31.06 | 152.85 |
| Kingaroy | Queensland | -26.53 | 151.84 |
| Lancelin | Western Australia | -31.01 | 115.33 |
| Lismore | New South Wales | -28.81 | 153.29 |
| Lowood | Queensland | -27.47 | 152.57 |
| Macksville | New South Wales | -30.72 | 152.92 |
| MacLean | Queensland | -27.8 | 153.02 |
| Maryborough | Queensland | -25.52 | 152.70 |
| Millmerran | Queensland | -27.88 | 151.27 |
| Mogo | New South Wales | -35.78 | 150.13 |
| Moree | New South Wales | -29.48 | 149.84 |
| Morgan | South Australia | -34.03 | 139.67 |
| Mount Barker | Western Australia | -34.63 | 117.67 |
| Muchea | Western Australia | -31.58 | 115.97 |
| Murray Bridge | South Australia | -35.13 | 139.27 |
| Nangiloc | Victoria | -34.47 | 142.35 |
| Narrabri | New South Wales | -30.33 | 149.78 |
| Neuarpurr | Victoria | -36.7 | 140.98 |
| Nundle/Hanging Rock | Victoria | -31.47 | 151.27 |
| Ongerup | Western Australia | -33.97 | 118.49 |
| Paruna | South Australia | -34.72 | 140.74 |
| Quilpie | Queensland | -26.62 | 144.27 |
| Rockley | New South Wales | -33.7 | 149.56 |
| St Arnaud | Victoria | -36.62 | 143.26 |
| Stockleigh | Queensland | -27.77 | 153.07 |
| Streaky Bay | South Australia | -32.8 | 134.21 |
| Sutton | New South Wales | -35.16 | 149.25 |
| Tamworth | New South Wales | -31.09 | 150.93 |
| Taree | New South Wales | -31.89 | 152.44 |
| Texas | Queensland | -28.85 | 151.17 |
| Tingha | New South Wales | -29.94 | 151.25 |
| Tintinara | South Australia | -35.88 | 140.05 |
| Toodyay | Western Australia | -31.55 | 116.45 |
| Tumbarumba | New South Wales | -35.77 | 148.01 |
| Walgett | New South Wales | -30.03 | 148.12 |
| Wanneroo | Western Australia | -31.75 | 115.82 |
| Warooka | South Australia | -34.99 | 137.40 |
| Warwick | Queensland | -28.22 | 152.03 |
| Wauchope | New South Wales | -31.47 | 152.73 |
| Wellstead | Western Australia | -34.6 | 118.75 |
| West Wyalong | New South Wales | -33.92 | 147.20 |
| Williamstown | South Australia | -34.67 | 138.89 |
| Woodburn | New South Wales | -29.08 | 153.36 |
| Wudinna | South Australia | -33.05 | 135.46 |
| Yanchep | Western Australia | -31.55 | 115.63 |
| Yarram | Victoria | -38.57 | 146.68 |
| Yeoval | New South Wales | -32.75 | 148.65 |
